# Temporal Factors and Missed Doses of Tuberculosis Treatment. A Causal Associations Approach to Analyses of Digital Adherence Data

**DOI:** 10.1513/AnnalsATS.201905-394OC

**Published:** 2020-04

**Authors:** Helen R. Stagg, James J. Lewis, Xiaoqiu Liu, Shitong Huan, Shiwen Jiang, Daniel P. Chin, Katherine L. Fielding

**Affiliations:** ^1^ Usher Institute of Population Health Sciences and Informatics, University of Edinburgh, Edinburgh, United Kingdom; ^2^ Department of Infectious Disease Epidemiology, London School of Hygiene & Tropical Medicine, London, United Kingdom; ^3^ National Center for Tuberculosis Control and Prevention, Chinese Center for Disease Control and Prevention, Beijing, China; ^4^ China Office, Bill & Melinda Gates Foundation, Beijing, China; and; ^5^ School of Public Health, University of the Witwatersrand, Johannesburg, South Africa

**Keywords:** adherence, compliance, tuberculosis, treatment

## Abstract

**Rationale:** Tuberculosis treatment lasts for 6 months or more. Treatment adherence is critical; regimen length, among other factors, makes this challenging. Globally, analyses mapping common types of nonadherence are lacking. For example, is there a greater challenge resulting from early treatment cessation (discontinuation) or intermittent missed doses (suboptimal dosing implementation)? This is essential knowledge for the development of effective interventions and more “forgiving” regimens, as well as to direct national tuberculosis programs.

**Objectives:** To granularly describe how patients take their tuberculosis medication and the temporal factors associated with missed doses.

**Methods:** The present study included patients with pulmonary tuberculosis enrolled in the control arm of a pragmatic, cluster-randomized trial in China of electronic reminders to improve treatment adherence. Treatment was the standard 6-month course (180 d), dosed every other day (90 doses). Medication monitor boxes recorded adherence (box opening) without prompting reminders. Patterns of adherence were visualized and described. Mixed-effects logistic regression models examined the temporal factors associated with per-dose suboptimal dosing implementation, adjusting for clustering within a participant. Cox regression models were used to examine the association between early suboptimal dosing implementation and permanent discontinuation.

**Results:** Across 780 patients, 16,794 (23.9%) of 70,200 doses were missed, 9,487 of which were from suboptimal dosing implementation (56.5%). By 60 days, 5.1% of participants had discontinued, and 14.4% had discontinued by 120 days. Most participants (95.9%) missed at least one dose. The majority of gaps were of a single dose (71.4%), although 22.6% of participants had at least one gap of 2 weeks or more. In adjusted models, the initiation–continuation phase transition (odds ratio, 3.07 [95% confidence interval, 2.68–3.51]) and national holidays (1.52 [1.39–1.65]) were associated with increased odds of suboptimal dosing implementation. Early-stage suboptimal dosing implementation was associated with increased discontinuation rates.

**Conclusions:** Digital tools provide an unprecedented step change in describing and addressing nonadherence. In our setting, nonadherence was common; patients displayed a complex range of patterns. Dividing nonadherence into suboptimal dosing implementation and discontinuation, we found that both increased over time. Discontinuation was associated with early suboptimal dosing implementation. These apparent causal associations between temporal factors and nonadherence present opportunities for targeted interventions.

Clinical trial registered with the ISRCTN Registry (ISRCTN46846388).

In 2017, 6.4 million incident tuberculosis (TB) cases were reported globally, and an estimated 3.6 million went undiagnosed or were not notified (1). Finding and treating these missing patients is a key target of the World Health Organization (WHO); this requires substantial international investment. It is critically important to protect this investment by providing effective treatment to every diagnosed patient.

The standard treatment for drug-sensitive TB lasts 6 months. Numerous studies have documented that patients struggle to adhere to the full course of therapy. An estimated 4–35% demonstrate poor adherence (2–11). Although various definitions have been used, poor adherence is associated with a reduced likelihood of sputum conversion (3), greater risk of an unsuccessful treatment outcome (4, 8, 12–15), and the development of drug resistance (16–19). Nonadherence to TB treatment is associated with various factors: those that are patient related, derived from the healthcare provider–patient relationship, the regimen itself, and the healthcare system (20).

In trials and observational studies, overly simplistic and non–evidence-based 80–90% adherence thresholds have traditionally been used to signify adequate adherence (12, 21–23). Recently, however, the importance of highly accurate means of measuring adherence within clinical trials has been acknowledged by WHO as a key part of trial design (24). Realistically, two core domains need to be considered when mapping adherence-persistence (time between first and last doses, capturing initiation and discontinuation) and dosing implementation (taking doses not as recommended; e.g., skipping weekends) (25). These components constitute “therapeutic coverage”: the proportion of time patients are exposed to efficacious drug concentrations (26). Detailed mapping of adherence patterns has been missing from the TB literature to date.

Knowledge of how exactly patients with TB take their medications and predictors of when nonadherence is most likely to occur is critical for the directed design of interventions to improve adherence, to develop regimens that are more “forgiving” of nonadherence, and to help clinicians know when to intervene with nonadherent patients. Currently, the relative burden of suboptimal dosing implementation and discontinuation is unknown globally; interventions to address these two components of nonadherence may look quite different. This is a critical knowledge gap regarding reducing the burden of nonadherence, which is impeding the most cost-effective implementation of the WHO guidelines on digital adherence technologies for TB treatment (27).

Using data collected from a trial of electronic reminders to improve medication adherence in China, we aimed to granularly describe how patients with TB take their medications and to determine if temporal factors were causally associated with missed doses to inform control efforts. Components of this study were reported previously in a conference abstract (28).

## Methods

### Parent Study and Study Population for Analysis

The parent study, a pragmatic, cluster-randomized trial of electronic reminders to improve treatment adherence among patients with pulmonary TB in the People’s Republic of China, from which these data are derived has been described before (*see* additional methods in the online supplement) (29). Participants were enrolled in the study between June 1, 2011, and March 7, 2012. Only participants in the control arm of the trial were included in this cohort study to capture usual patterns of treatment adherence in the absence of an intervention (*see* additional methods in the online supplement).

### Measuring and Defining Adherence to Treatment

Adherence to each dose of treatment was documented by a medication monitor box (*see* additional methods in the online supplement). The box captured every date and time that it was opened; box opening did not necessarily mean that drugs were taken. Medication was dosed every other day (as per the National TB Program [NTP] standard at the time) for 90 doses over a 180-day period. If the box was opened at least once within each 2-day dosing window, this was recorded as adherence. The standard 6-month regimen for drug-sensitive TB was used (2 mo of isoniazid, rifampicin, ethambutol, and pyrazinamide, followed by 4 mo of isoniazid and rifampicin). Medication was not dosed in combination pills.

Nonadherence data from the monitor were coded and categorized as a dose missed because of suboptimal dosing implementation versus a dose missed because of permanent discontinuation, using accepted terminology as per Vrijens and colleagues (25). “Discontinuation” was defined as ceasing to adhere to treatment and not recommencing both *1*) at any point during the 180-day period and *2*) after this period but before the end of the trial. Discontinuation is different from the programmatically defined term “lost to follow-up” (previously known as “default”), when either a patient’s treatment is interrupted for two consecutive months or more or a patient does not start treatment. “Suboptimal dosing implementation” refers to all doses missed during the 180-day period, aside from those due to discontinuation. The term “suboptimal” is not intended to imply a judgment about the appropriate degree of adherence/type of adherence pattern required to achieve a positive treatment outcome; rather, it reflects an implementation level below 100% of doses taken.

### Temporal Exposures and Potential Confounders

The following temporal measures were calculated from the medication monitor data: *1*) day of the week, *2*) treatment month, *3*) whether the dose fell on a Chinese national holiday, and *4*) whether the patient was in the initiation or continuation phase of treatment (*see* additional methods in the online supplement). In addition, data were available for a series of potential confounders, all of which were self-reported at entry into the study. These included age, sex, marital status, education level, occupation, household income, type of medical insurance, registration status, and distance from home to TB clinic. The county/district in which the participant lived was grouped as broadly rural or urban.

### Statistical Methods

#### Descriptive analyses

Analyses were undertaken using Stata 15 software (StataCorp), and graphs were plotted using Excel software (Microsoft Corp.). Adherence to treatment was described using the following summary measures: overall percentage of doses taken, average duration that a patient was receiving treatment before ceasing completely, percentage of participants achieving an 80% adherence threshold, and percentage of participants achieving a 90% threshold. To account for clustering, for each measure, the mean was calculated per county/district, and then the geometric mean was taken across the county/district values. Adherence over time, grouped by different percentage intervals, was graphically visualized using lasagna plots in which white indicates nonadherence (30).

Line graphs were used to visualize nonadherence due to suboptimal dosing implementation versus permanent discontinuation from treatment for all participants in the study and by adherence levels in the initiation phase (31). After plotting these graphs, we decided to separate suboptimal dosing implementation and discontinuation in the remaining analyses. The length and number of gaps in treatment due to suboptimal dosing implementation were described using scatterplots.

#### Associations between temporal factors and suboptimal dosing implementation

We used mixed-effects logistic regression to examine the factors associated with nonadherence due to suboptimal dosing implementation, treating each dose as an observation and adjusting for clustering within a individual. We focused on temporal factors, including weekends, national holidays, and the initiation–continuation phase transition (Model 1) or treatment months (Model 2). Our methodology, including details of model selection through the use of directed acyclical graphs, determination of *a priori* confounders, and assessment of potential effect modification, is detailed elsewhere (*see* additional methods in the online supplement). The impact of using different confounder sets on our findings was explored through Models 1A–1F (*see* additional methods in the online supplement). Both approaches sought to address all confounding using different confounder sets to support the drawing of causal conclusions from observational data (32). The potential presence of an interaction between the three temporal factors weekends, national holidays, and the initiation–continuation phase transition and *1*) county/district or *2*) distance from home to TB clinic was also explored using likelihood ratio tests (LRTs) (Models 1G and 1H).

#### Associations between early suboptimal dosing implementation and time to discontinuation

Cox proportional hazards regression was used to assess whether early suboptimal dosing implementation, either in the initiation phase (Model 3) or in Month 1 (Model 4), was associated with time to discontinuation. Individuals who had discontinued in the initiation phase and Month 1 were excluded, respectively, to preserve the temporality of the association. Further details on adjustment for confounding and so forth are presented in the additional methods in the online supplement. We report the results of sensitivity analyses on the impact of confounding by county/district (Models 3F and 4F) and excluding individuals who discontinued during the last three doses of treatment (Models 3G and 4G). The potential presence of an interaction between early suboptimal dosing and *1*) county/district or *2*) distance from home to the TB clinic was also explored using LRTs.

### Ethical Approval

The trial was approved by the ethics committees of the Chinese Center for Disease Control and Prevention (201008) and the London School of Hygiene & Tropical Medicine (5704). All participants provided written consent before inclusion in the trial.

## Results

### Characteristics of the Study Population

Of the 1,104 individuals randomized to the control arm of the trial, 209 (18.9%) had technical issues with the medication monitor because of power outage problems, as indicated by the box resetting the date to a baseline value (*see* Figure E1 in the online supplement). A further 10.4% of patients (*n* = 115) were excluded because events such as hospitalization for more than 3 days removed the potential for treatment to be monitored for the entire period. Thus, data of 780 (70.7%) patients were available for analysis. A comparison of the included and excluded patients revealed similarity in terms of baseline characteristics, except for county/district and distance from home to the TB clinic (Table E1).

The baseline characteristics of participants are presented in Table 1. Individuals were generally male (*n* = 535; 68.6%). More than half were under the age of 50 years (*n* = 525; 67.3%). Farming was the largest occupation (*n* = 384; 49.2%), with 516 (66.2%) individuals living in counties/districts deemed rural and 500 (64.1%) ensured through rural cooperatives.

**Table 1.** Baseline characteristics and unadjusted analyses of factors associated with nonadherence due to suboptimal dosing implementation or discontinuation

Exposure Variables	Overall		Analysis of Suboptimal Dosing Implementation					Analysis of Discontinuation										
	Participants	Column %	Doses	Column %	Doses Missed	Row %	Unadjusted OR (95% CI)	Analysis Time (Doses)	Participants Who Discontinued	Unadjusted HR (95% CI)								
Overall	780	100.0	62,893	100.0	9,487	15.1	—	62,396	235	—								
Sex																		
Female	245	31.4	19,804	31.5	2,683	13.5	Baseline	19,649	161	Baseline								
Male	535	68.6	43,089	68.5	6,804	15.8	1.20 (0.99–1.45)	42,747	74	1.00 (0.76–1.32)								
Age category, yr																		
<30	230	29.5	18,305	29.1	2,837	15.5	Baseline	18,157	69	Baseline								
30–39	128	16.4	10,099	16.1	1,315	13.0	1.01 (0.95–1.08)	10,021	44	0.95 (0.87–1.04)								
40–49	167	21.4	13,518	21.5	2,077	15.4		13,422	56									
50–59	136	17.4	11,117	17.7	1,712	15.4		11,023	35									
60+	119	15.3	9,854	15.7	1,546	15.7		9,773	31									
Occupation																		
Student	32	4.1	2,529	4.0	428	16.9	1.01 (0.64–1.58)	2,512	13	1.34 (0.76–2.37)								
Worker	74	9.5	6,102	9.7	722	11.8	0.61 (0.45–0.84)	6,048	17	0.69 (0.42–1.15)								
Migrant worker	74	9.5	6,167	9.8	815	13.2	0.76 (0.55–1.03)	6,115	17	0.68 (0.41–1.13)								
Farmer	384	49.2	30,763	48.9	5,347	17.4	Baseline	30,523	122	Baseline								
Unemployed/houseworker	63	8.1	5,207	8.3	624	12.0	0.60 (0.43–0.84)	5,165	17	0.81 (0.49–1.35)								
Other	153	19.6	12,125	19.3	1,551	12.8	0.68 (0.53–0.86)	12,033	49	1.02 (0.73–1.42)								
Education level																		
Illiterate	60	7.7	4,595	7.3	858	18.7	1.38 (0.92–2.07)	4,557	20	1.43 (0.79–2.59)								
Lower middle school	494	63.3	39,999	63.6	6,254	15.6	1.03 (0.78–1.35)	39,692	154	1.25 (0.82–1.93)								
Upper middle school	130	16.7	10,571	16.8	1,216	11.5	0.73 (0.52–1.02)	10,484	37	1.13 (0.68–1.89)								
University or more	96	12.3	7,728	12.3	1,159	15.0	Baseline	7,663	24	Baseline								
Total household income in last calendar yr (RMB)																		
≥20,000	446	57.2	36,044	57.3	4,994	13.9	Baseline	35,754	131	Baseline								
<20,000	334	42.8	26,849	42.7	4,493	16.7	1.31 (1.10–1.57)	26,642	104	1.07 (0.83–1.38)								
Medical insurance																		
Rural cooperative	500	64.1	40,583	64.5	6,604	16.3	1.23 (0.96–1.57)	40,261	146	0.65 (0.48–0.89)								
Urban workers	92	11.8	7,843	12.5	946	12.1	0.86 (0.61–1.20)	7,773	18	0.40 (0.24–0.69)								
No insurance	132	16.9	9,855	15.7	1,350	13.7	Baseline	9,786	53	Baseline								
Other	56	7.2	4,612	7.3	587	12.7	0.95 (0.64–1.41)	4,576	18	0.71 (0.42–1.21)								
Marital status																		
First marriage	551	70.6	45,024	71.6	6,707	14.9	Baseline	44,665	161	Baseline								
Unmarried	184	23.6	14,421	22.9	2,194	15.2	1.02 (0.82–1.26)	14,305	57	1.11 (0.82–1.50)								
Other	45	5.8	3,448	5.5	586	17.0	1.15 (0.78–1.69)	3,426	17	1.43 (0.87–2.35)								
County																		
Baiquan	100	12.8	7,629	12.1	1,926	25.2	Baseline	7,581	46	Baseline								
Yilan	103	13.2	8,683	13.8	1,113	12.8	0.40 (0.29–0.56)	8,605	15	0.26 (0.15–0.47)								
Rugao	78	10.0	6,366	10.1	844	13.3	0.39 (0.27–0.56)	6,310	21	0.52 (0.31–0.87)								
Jianhu	80	10.3	6,938	11.0	1,270	18.3	0.60 (0.42–0.86)	6,878	13	0.29 (0.16–0.54)								
Miluo	85	10.9	6,961	11.1	1,131	16.2	0.55 (0.39–0.78)	6,905	24	0.55 (0.34–0.90)								
Yueyanglou	81	10.4	5,893	9.4	718	12.2	0.35 (0.24–0.50)	5,856	42	1.21 (0.79–1.83)								
Fengjie	70	9.0	5,115	8.1	684	13.4	0.39 (0.27–0.56)	5,088	40	1.34 (0.88–2.04)								
Shapingba	79	10.1	6,311	10.0	915	14.5	0.49 (0.34–0.70)	6,258	18	0.45 (0.26–0.78)								
Jiangbei	104	13.3	8,997	14.3	886	9.8	0.29 (0.21–0.40)	8,915	16	0.27 (0.16–0.49)								
Rural/urban																		
Rural	516	66.2	41,692	66.3	6,968	16.7	Baseline	41,367	159	Baseline								
Urban	264	33.8	21,201	33.7	2,519	11.9	0.67 (0.55–0.81)	21,029	76	0.94 (0.71–1.23)								
Residence																		
Living in place of household registration	658	84.4	53,187	84.6	8,191	15.4	Baseline	52,768	198	Baseline								
Not living in place of household registration	122	15.6	9,706	15.4	1,296	13.4	0.80 (0.62–1.03)	9,628	37	1.03 (0.72–1.46)								
Distance from home to local TB clinic, km																		
<10	188	24.1	15,984	25.4	2,255	14.1	Baseline	15,847	37	Baseline								
10–19	191	24.5	15,185	24.1	2,199	14.5	1.05 (0.99–1.12)	15,065	63	1.14 (1.04–1.24)								
20–29	118	15.1	9,596	15.3	1,451	15.1		9,524	40	—								
30–39	149	19.1	11,802	18.8	1,782	15.1		11,714	49	—								
≥40	134	17.2	10,326	16.4	1,800	17.4		10,246	46	—								
Day							*P* < 0.001											
Sunday	—	—	9,009	14.3	1,516	16.8	Baseline	—	—	—								
Monday	—	—	8,997	14.3	1,301	14.5	0.81 (0.74–0.89)	—	—	—								
Tuesday	—	—	8,939	14.2	1,344	15.0	0.84 (0.77–0.92)	—	—	—								
Wednesday	—	—	9,004	14.3	1,315	14.6	0.83 (0.76–0.91)	—	—	—								
Thursday	—	—	8,895	14.1	1,426	16.0	0.93 (0.85–1.01)	—	—	—								
Friday	—	—	9,275	14.7	1,251	13.5	0.74 (0.68–0.81)	—	—	—								
Saturday	—	—	8,774	14.0	1,334	15.2	0.87 (0.80–0.95)	—	—	—								
Weekend							*P* < 0.001											
Weekday	—	—	45,110	71.7	6,637	14.7	Baseline	—	—	—								
Weekend	—	—	17,783	28.3	2,850	16.0	1.13 (1.07–1.19)	—	—	—								
Month							*P* < 0.001											
1	—	—	11,687	18.6	789	6.8	Baseline	—	—	—								
2	—	—	11,298	18.0	1,383	12.2	2.92 (2.60–3.28)	—	—	—								
3	—	—	10,800	17.2	1,857	17.2	5.35 (4.66–6.13)	—	—	—								
4	—	—	10,314	16.4	1,843	17.9	5.78 (4.91–6.81)	—	—	—								
5	—	—	9,770	15.5	1,839	18.8	6.31 (5.21–7.65)	—	—	—								
6	—	—	9,024	14.3	1,776	19.7	6.26 (5.01–7.83)	—	—	—								
National holidays							*P* < 0.001											
No	—	—	58,018	92.2	8,487	14.6	Baseline	—	—	—								
Yes	—	—	4,875	7.8	1,000	20.5	1.62 (1.49–1.75)	—	—	—								
Phase							*P* < 0.001											
Initiation	—	—	22,985	36.5	2,172	9.4	Baseline	—	—	—								
Continuation	—	—	39,908	63.5	7,315	18.3	3.09 (2.70–3.54)	—	—	—								
Initiation phase adherence*										*P* = 0.003								
≥90%	—	—	—	—	—	—	—	47,419	137	Baseline								
80–90%	—	—	—	—	—	—	—	7,373	22	1.05 (0.67–1.64)								
<80%	—	—	—	—	—	—	—	6,819	36	1.98 (1.37–2.86)								
Month 1 adherence^†^										*P* = 0.003								
≥90%	—	—	—	—	—	—	—	51,106	171	Baseline								
80–90%	—	—	—	—	—	—	—	7,471	34	1.39 (0.96–2.00)								
<80%	—	—	—	—	—	—	—	3,757	25	2.10 (1.38–3.19)								

*Definition of abbreviations*: — = not applicable; CI = confidence interval; HR = hazard ratio; OR = odds ratio; RMB = renminbi; TB = tuberculosis.

Leftmost data columns: baseline characteristics of the 780 individuals from the control arm of the original trial. Middle data columns: unadjusted mixed-effects logistic regression for the 780 individuals included in the analysis of suboptimal dosing implementation. Each model was adjusted for clustering within a patient. Age and distance to TB clinic were modeled as linear variables. Random effects were modeled on the initiation–continuation phase and month variables within the relevant unadjusted model. Rightmost data columns: unadjusted Cox regression for the 780 individuals included in the analysis of discontinuation. Age and distance to TB clinic were modeled as linear variables. Analysis time documents the time at risk and under observation. All columns: No data were missing for any of the variables.

* A total of 740 individuals were included in the initiation phase adherence model; this exposure variable documents nonadherence due to suboptimal dosing implementation only.

^†^ A total of 775 individuals were included in the Month 1 adherence model; this exposure variable documents nonadherence due to suboptimal dosing implementation only.

### Summary Measures of Overall Adherence

Across all 780 study participants, 70,200 doses were scheduled during the 180-day period; 16,794 of these were missed (23.9%). The geometric mean number of doses taken was 68/90 (75.6%). The geometric mean duration of receiving treatment was 80 doses (i.e., 160 d) before discontinuation.

### Overall Adherence over Time

**Figure 1.**
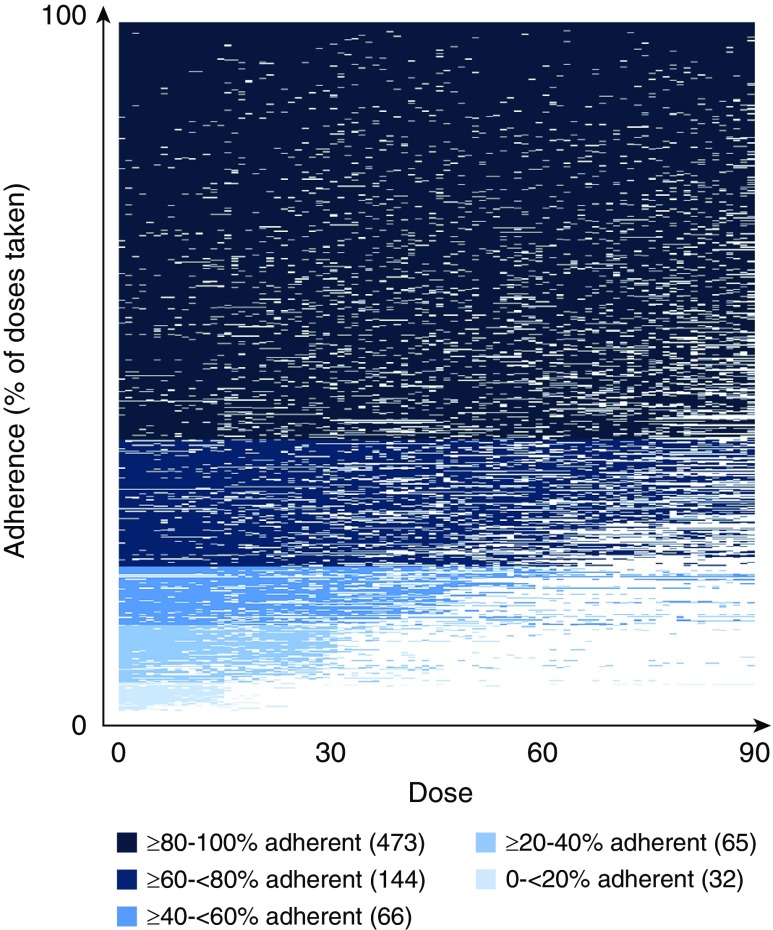
Lasagna plot of adherence. Each patient of the 780 participants in the control arm of the original trial is represented by a row in the figure; white indicates a dose that has not been taken. Adherence was calculated as a percentage of the 90 doses taken over the 180-day period and then grouped into 20% adherence intervals. Rows are colored by adherence group. Numbers in brackets indicate the number of individuals within each 20% adherence interval. Reprinted by permission from Reference 28.

Lasagna plots of adherence over time demonstrated the distribution of participants in 20% adherence intervals, with 473 (60.6%) of 780 in the highest category of greater than or equal to 80% to 100% adherent (Figure 1). A clear “staggered” pattern was observed in the lowest categories that corresponded to dropoffs in adherence with each passing month (15 doses; 30 d). Although there was a reduction in adherence over time, erratic nonadherence (suboptimal dosing implementation) was observed throughout the treatment period.

**Figure 2.**
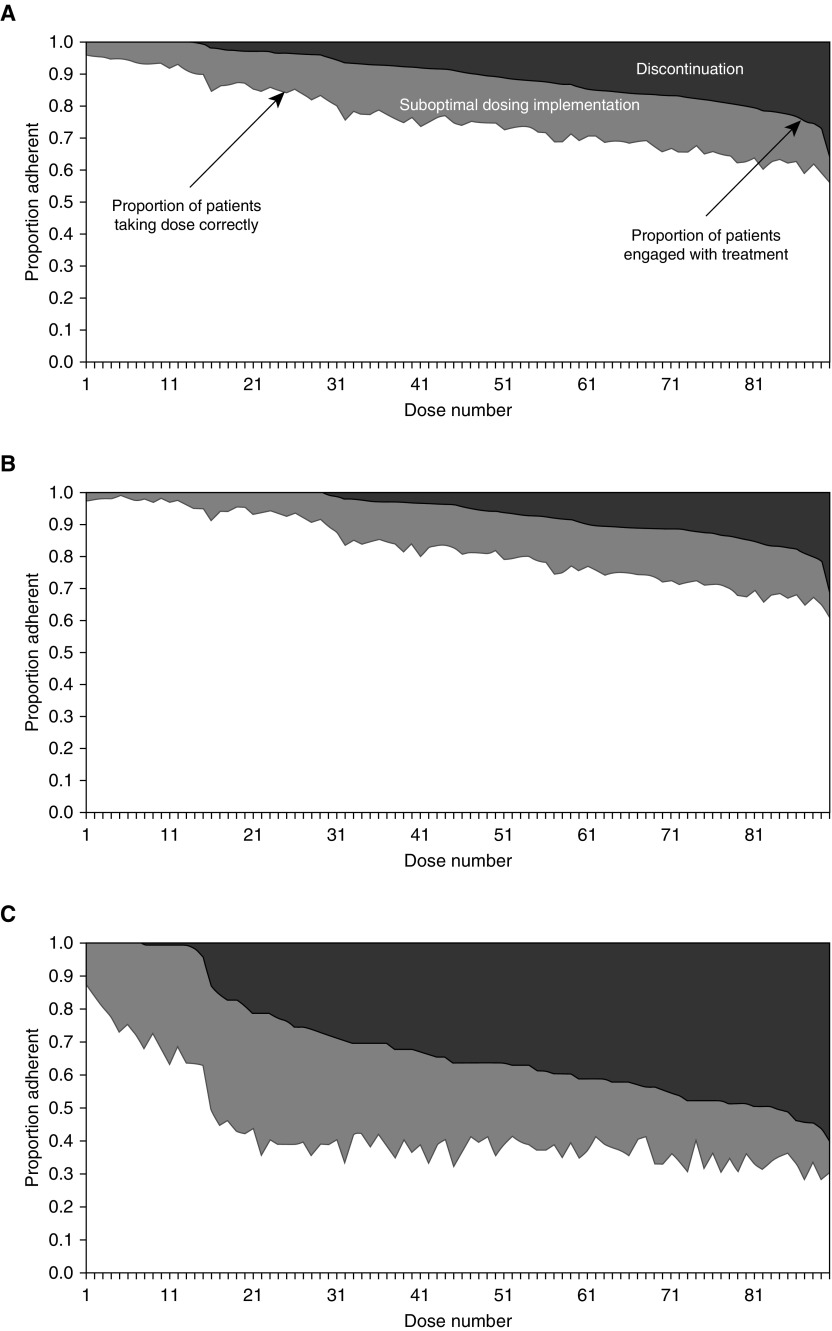
Relative contribution of discontinuation and suboptimal dosing implementation to nonadherence over time. Nonadherence due to discontinuation (ceasing treatment and not recommencing; dark gray) versus suboptimal dosing implementation (sporadic missed doses; light gray) over time in (*A*) the 780 control arm patients from the original trial, (*B*) the 659 patients who displayed greater than or equal to 80% adherence during the initiation phase, and (*C*) the 121 patients who displayed less than 80% adherence in the initiation phase. Discontinuation is ceasing treatment at any stage, including only for the 90th dose. If, after the 90th dose, another dose was taken before the end of the trial, the patient is not recorded as having discontinued. Discontinuation is not the same as programmatically defined loss to follow-up/default. Figure style adapted from Reference 31.

The relative importance of nonadherence due to the permanent discontinuation of treatment versus suboptimal dosing implementation is shown in Figure 2A. Of the 16,794 missed doses, 9,487 were due to suboptimal dosing implementation (56.5%), and the remainder were due to discontinuation. The impact of discontinuation was demonstrably stronger over time. By the end of Month 2, 5.1% of individuals had discontinued treatment; this figure was 14.4% by the end of Month 4 and continued to increase during the last two months until it reached 36.3% at the end of the 180-day period. The latter figure reflects the fact that discontinuation captures treatment cessation without recommencement at any time point, including cessation at the last (90th) dose. When the 121 participants with less than 80% adherence in the initiation phase were examined separately, they demonstrated sharp and sustained reductions in adherence due to both discontinuation and suboptimal dosing implementation (Figure 2C).

### Gaps in Adherence (Suboptimal Dosing Implementation)

**Figure 3.**
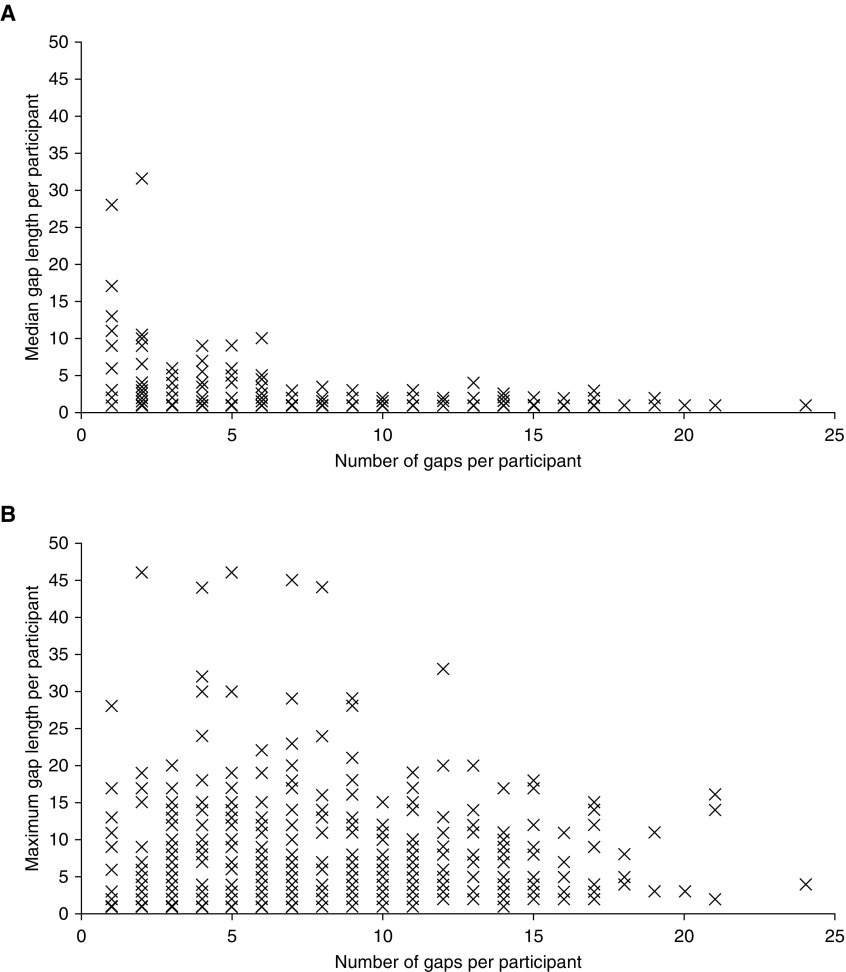
Gaps in adherence. Gaps during the 90-dose medication period among the 748 participants who displayed suboptimal dosing implementation. Number of gaps per participant of any length plotted against (*A*) the median gap length per participant and (*B*) the maximum gap length per participant.

Suboptimal dosing implementation was demonstrated by 748 (95.9%) of 780 participants; that is, they displayed at least one gap in their treatment of one dose or more that was not due to discontinuation. Overall, a total of 4,677 gaps were recorded, of which 71.4% (3,337 of 4,677) were for one dose only. The population median of the median gap length per participant was 1, and the interquartile range was 1–1 (Figure 3A). When the maximum gap length per participant was examined, the median across the population was two doses (interquartile range, 1–6) (Figure 3B). Of the 780 individuals, 368 (47.2%) had at least one gap of three doses (roughly 1 wk) or more, and 176 (22.6%) had at least one gap of seven doses (roughly 2 wk) or more.

### Associations between Suboptimal Dosing Implementation and Temporal Factors

Our analysis of suboptimal dosing implementation and temporal factors was composed of 780 patients and 62,893 dose observations (Table 1). In unadjusted analyses, a strong association was seen between the initiation–continuation phase transition and suboptimal dosing implementation. The continuation phase was associated with triple the odds of suboptimal dosing implementation (odds ratio, 3.09 [95% confidence interval, 2.70–3.54]). This mirrors the month-by-month findings, in which suboptimal dosing implementation increased from 6.8% of doses in Treatment Month 1 to 19.7% in Treatment Month 6. Sunday was associated with greater suboptimal dosing implementation than the other days of the week (*P* < 0.001). Compared with weekdays, weekends were associated with a small increase in the odds of suboptimal dosing implementation (1.13 [1.07–1.19]). National holidays were associated with a larger increase in odds of suboptimal dosing implementation (1.62 [1.49–1.75]; 14.6–20.5%).

In an adjusted model controlling for age as a linear variable, as well as sex and urban versus rural setting, and with a random effect on the initiation–continuation variable (LRT *P* < 0.001), all three temporal variables were associated with greater odds of suboptimal dosing implementation (weekends, 1.14 [1.08–1.20]; national holiday, 1.52 [1.39–1.65]; initiation–continuation transition, 3.07 [2.68–3.51]) (Model 1). There was no evidence for interactions between the initiation–continuation transition and national holidays (LRT *P* = 0.97) or weekends (LRT *P* = 0.07). These findings were robust to adjustment for different combinations of confounders (Table E2, Models 1A–1F).

Tests for interaction were performed between the three temporal factors and county/district or distance. For distance, the LRT *P* values for the initiation–continuation phase transition, holidays, and weekends were 0.52, 0.97, and 0.91, respectively. For county/district, the LRT *P* values for the initiation–continuation phase transition, holidays, and weekends were 0.01, less than 0.001, and 0.79, respectively. We thus undertook stratified analyses by county/district of the relationship between suboptimal implementation and *1*) the initiation–continuation phase transition (Table E3, Model 1G) or *2*) holidays (Table E4, Model 1H). Although the magnitude of the relationship between these two temporal factors and suboptimal implementation was altered by county, the direction of effect was the same in all instances, barring one instance in which the confidence interval crossed the null (Baiquan County, Model 1H; 0.94 [0.75–1.18]).

Given the striking initiation–continuation phase effect found in these models, but also the more gradual pattern of reducing adherence demonstrated in Figure 1, the association between treatment month and suboptimal dosing implementation was assessed. A random effect was included on the treatment month (LRT *P* < 0.001), which was treated as a categorical variable. An interaction was documented between treatment month and national holidays (LRT *P* = 0.01), but the statistical evidence was less certain for an interaction between treatment month and weekends (LRT *P* = 0.06).

Within a model containing the treatment month–national holiday interaction (Model 2), the association between weekends and the odds of nonadherence due to suboptimal dosing implementation changed little from Model 1 (1.14 [1.08–1.20]). From month to month, the likelihood of suboptimal dosing implementation approximately increased and was particularly pronounced for doses that fell on national holidays (Table 2). A dose falling on a national holiday was positively associated with suboptimal dosing implementation, with the largest increase in odds in the last month of treatment, but no clear trend from month to month (Table 2).

**Table 2.** Adjusted odds ratios for the association between suboptimal dosing implementation and treatment month, stratified by national holidays, or national holidays, stratified by treatment month

	National Holidays	
	No	Yes
Treatment month, stratified by national holidays		
Treatment month		
1	Baseline	Baseline
2	2.87 (2.55–3.23)	3.32 (2.15–5.15)
3	5.23 (4.55–6.01)	5.82 (3.81–8.90)
4	5.58 (4.72–6.58)	7.34 (4.76–11.31)
5	6.23 (5.13–7.57)	6.45 (4.11–10.12)
6	5.90 (4.71–7.40)	10.01 (6.27–15.98)
National holidays, stratified by treatment month		
Treatment month		
1	Baseline	1.25 (0.85–1.84)
2	Baseline	1.45 (1.15–1.82)
3	Baseline	1.39 (1.16–1.67)
4	Baseline	1.64 (1.36–1.98)
5	Baseline	1.29 (1.06–1.58)
6	Baseline	2.12 (1.71–2.62)

The table shows the results of adjusted regression of the association between nonadherence due to suboptimal dosing implementation and treatment month, stratified by national holidays (top rows), or national holidays, stratified by treatment month (bottom rows). Model 2: A total of 62,893 doses from 780 individuals from the control arm of the original trial are included. The stratum-specific odds ratios are adjusted for weekends, age, sex, and rural versus urban setting. Random effects are modeled on the month variable. Age is modeled as a linear variable. Results per cell are presented as odds ratio (95% confidence interval).

### Associations between Time to Discontinuation and Early Suboptimal Dosing Implementation

Among the individuals included in the study, 109 were found to stop treatment without recommencing within the 90-dose period but to later recommence before the end of the trial. The latest dose taken was at 254 days. These individuals were not classified as discontinuing. Patients who discontinued during the relevant implementation period were excluded to preserve temporality within any associations. Thus, 740 patients contributed to an analysis of discontinuation and suboptimal dosing implementation in the initiation phase, and 775 contributed when suboptimal dosing implementation in Month 1 was instead considered (Table 1).

In unadjusted analyses, increased suboptimal dosing implementation in the initiation phase and Month 1 was associated with an increase in the likelihood of discontinuation (Table 1). These findings were robust in an adjusted analysis (Table 3). The impact of greater than or equal to 80% to less than 90% adherence versus greater than or equal to 90% adherence was less certain for the initiation phase analysis (Model 3), but it was more suggestive of a dose–response association in the Month 1 analysis (Model 4). Considering different confounder sets, these models were robust to adjustment for a fixed effect for county/district rather than urban/rural (Table E5, Models 3F and 4F). When the 52 individuals who discontinued from dose 87 onward were excluded, our effect estimates increased for both the initiation phase and Month 1 analyses (Table E5, Models 3G and 4G). Tests for interaction between early suboptimal dosing implementation and county/district revealed no evidence for an effect (LRT *P* = 0.19).

**Table 3.** Adjusted Cox regression models of the association between early suboptimal dosing implementation and discontinuation

Temporal Factor	Hazard Ratio (95% CI)
Model 3	
Initiation phase adherence	*P* = 0.004
≥90%	Baseline
80% to <90%	1.04 (0.66–1.63)
<80%	1.97 (1.36–2.85)
Model 4	
Month 1 adherence	*P* = 0.004
≥90%	Baseline
80% to <90%	1.37 (0.95–1.99)
<80%	2.06 (1.35–3.15)

*Definition of abbreviation*: CI = confidence interval.

Model 3 examines the association between nonadherence in the initiation phase due to suboptimal dosing implementation and discontinuation, adjusting for age, sex, and rural versus urban setting. It excludes individuals who discontinued in the initiation phase, leaving 740. Model 4 examines the association between nonadherence in Month 1 due to suboptimal dosing implementation and discontinuation, adjusting for age, sex, and rural versus urban setting. It excludes individuals who discontinued during Month 1, leaving 775. Age was modeled as a linear variable.

## Discussion

Our analysis of adherence, both suboptimal dosing implementation and discontinuation, among patients with pulmonary TB in China provides the first detailed description of how doses are missed over a 6-month treatment period. We found that participants took 76% of their doses; 61% took 80% or more. The use of simple percentage thresholds, however, masks important variation in the patterns of missed doses over time.

Of all missed doses, 43% were due to discontinuation. A steady increase was observed in nonadherence due to both suboptimal dosing implementation and discontinuation over time. At 2 months, 5.1% of participants had discontinued their medication; 14.4% had discontinued at 4 months, and 36.3% had discontinued by the end of the 180-day period. During the intensive phase of treatment (the first 2 mo), suboptimal dosing implementation accounted for the majority of nonadherence. Of the 19% of patients who were nonadherent at the end of the intensive phase, discontinuation accounted for 27% of the nonadherence, and suboptimal dosing implementation accounted for the remainder. During the continuation phase (Months 3–6), the odds of suboptimal dosing implementation were three times higher than during the intensive phase, but the percentage of patients with suboptimal dosing implementation remained stable at 17–20%. However, the percentage of those who discontinued treatment continued to accumulate, and by the fifth month, discontinuation accounted for 52% of all nonadherence.

We identified an important association between suboptimal dosing implementation early in the course of treatment and subsequent discontinuation. Suboptimal dosing implementation in the first month or overall initiation phase (Months 1 and 2) was associated with higher discontinuation rates. Across participants, 96% demonstrated suboptimal dosing implementation; approximately three-fourths of gaps were for one dose only. Nevertheless, 47% of individuals had potentially clinically important gaps of three consecutive doses or more, and 23% had potentially clinically important gaps of seven consecutive doses (about 2 wk) or more. The odds of suboptimal dosing implementation were higher on national holidays (odds ratio, 1.52).

The findings of this study provide several insights into how drug-sensitive TB treatment can be improved. First, NTPs should take seriously the problem of nonadherence to treatment, which is underrecognized. In this study, a high percentage of patients had gaps of 1 week or more in their treatment due to suboptimal dosing implementation. If these gaps are not recognized and treatment is not adjusted accordingly, then long-term relapse-free cure of these patients may be compromised. NTPs should place a much higher priority on improving adherence during treatment and not simply focus on ensuring completion.

Second, this study identified the importance of early adherence. Adherence worsened over the course of treatment, especially after the shift into the continuation phase. We also found an association between discontinuation and early suboptimal dosing implementation. Thus, improving adherence early in the course of treatment may be important to prevent later nonadherence.

Third, this study highlights the importance of granular adherence data on individual patients. Early identification of individuals with poor adherence or who discontinue would improve the likelihood of success of adherence-promoting interventions. Identification of such individuals could result in the initiation of differentiated care, which would include more tailored adherence support for these patients. The design of such behavioral interventions should take into account data on the types of nonadherence displayed by the target population and their causes. For example, plans to support medication adherence may need to be generated proactively with patients before holiday periods, when travel to different locations may generate greater concern about stigma and result in missed doses. In order to check for improvement, adherence should also be monitored after such interventions are deployed. Digital technologies to record adherence, such as by using pill bottle opening as a surrogate for medication intake, have been available for many years and are starting to be rolled out globally, despite operational barriers such as cost (33). Such technologies, however, provide an opportunity to monitor TB treatment adherence in individual patients on a large scale (33).

Fourth, these results lend support to the development of shorter treatment regimens, which may avoid the adherence dropoff later in treatment that is currently observed. Such regimens have not yet demonstrated noninferiority (34–36); however, they will likely increase the importance of each individual dose in ensuring cure. Retrieving patients who default from treatment is a large financial burden on NTPs; this could also be reduced with shorter regimens that result in less discontinuation. We also highlight the value of examining discontinuation of treatment, rather than programmatically defined loss to follow-up/default, in terms of capturing effective drug exposure.

Overall, previous studies have provided the initial basis of a link between different adherence patterns and treatment outcomes in drug-sensitive disease (2–9, 11). For example, missing 8–16% of doses has been associated with 25 times the odds of remaining sputum positive (3), adhering below a 90% threshold with 5.9 times the rate of an unfavorable outcome (15), adhering below a 75% threshold with 3.2 times the odds of recurrence (14), adhering below a 90% threshold with 3.4 times the odds of mortality (4), and “irregular” drug taking such that treatment had to be extended with 2.5 times increased odds of relapse (8). Conversely, a regimen simulating less than 67% adherence had no impact on recurrence (37). In addition, previous studies have documented a 17% additional hazard per month of acquired drug resistance if adherence is less than 80% (19), or 19.7 times the odds with half-month gaps, nonengagement, or less than 80% adherence (16). This association is not simple; particularly poor adherence may exert little selective pressure (17). In drug-resistant disease, there is a smaller but less contradictory evidence base in terms of the implications of nonadherence: Long interruptions and less than 80% to 90% adherence have been associated with poorer outcomes (17, 19, 38, 39). What these studies lack, which potentially explains their conflicting findings, is a granular exploration of how nonadherence influences treatment outcomes using reliable sources of adherence data (23). Our study indicates that poor adherence is complicated and heterogeneous; future studies will require granular dose-by-dose data to properly assess the nonadherence–outcome relationship. Future studies should collect detailed adherence data, moving away from monthly self-reported information and chart reviews, to ascertain how they correlate to therapeutic coverage, pharmacokinetics (TB drugs with a short half-life are predicted to be less forgiving), sputum conversion rates, treatment outcomes (40), relapse (the gold standard outcome measure), and the development of drug resistance.

This is the most detailed analysis to date of treatment adherence in TB that makes use of exceptionally granular adherence data. It does, however, have its limitations. Whether drug intake was supported (e.g., observed by a family member) or self-administered was not documented, potentially leaving residual confounding. Opening the medication monitor box does not necessarily mean that drugs were taken, although a validation study has indicated high correlation with urine rifampicin concentrations (41). Given that each dose could have been taken during a 2-day period, nondifferential misclassification of the temporal exposure variables may have occurred, biasing effect estimates toward the null. Fixed-dose combination pills were not used, so it is possible that nonadherence was underestimated per drug, because individuals may have chosen not to take all of their pills per dose. The exclusion of participants for whom a whole dosing history was not available may have resulted in selection bias, because excluded participants differed from included participants in terms of the county/district in which they lived and their distance from home to their local TB clinic. On the basis of tests for interaction, it seems unlikely, however, that temporal factors (the focus of our analysis) are systematically differently associated with adherence across different levels of these variables. Data were missing on participants’ personal holidays, which could bias the effect size toward the null. Furthermore, part of the national holiday effect could represent individuals not transporting their monitor boxes with them when they travel, but nevertheless taking their medication. Sociobehavioral data on factors associated with nonadherence, such as stigma, were not collected, potentially resulting in residual confounding. Finally, participants may have been aware that they would be less likely to have taken their drugs on weekends and thus switched their doses from weekends to weekdays to avoid nonadherence. This is a function of the every-other-day dosing of the regimen and would result in an overemphasized effect size.

Four key factors in our study affect generalizability: This was a *1*) single-country dataset of *2*) patients with pulmonary TB *3*) enrolled in a trial who *4*) took their drugs every other day. Being enrolled in a trial is believed to boost adherence, and the individuals who consent to participate are often more likely to be adherent; adherence data from observational studies globally are therefore also needed (42–44). We thus recommend future studies using granular adherence data from observational studies undertaken in other nations.

### Conclusions

In conclusion, we demonstrate how nonadherence to TB treatment is a complex issue that needs to be taken seriously. Adherence worsens over the course of treatment, but early-stage interventions (when suboptimal dosing implementation is first detected) may prevent later discontinuation. For such interventions to be accurately targeted to the patients most in need, individual-level adherence data are required on a large scale. Shorter TB treatment regimens may reduce the impact of worsening adherence over the treatment course.

## Supplementary Material

Supplements

Author disclosures
