# Supplementary material for: Temporal Factors and Missed Doses of Tuberculosis Treatment. A Causal Associations Approach to Analyses of Digital Adherence Data
Source: Ann Am Thorac Soc. 2020 Apr;17(4):438–49. doi: 10.1513/AnnalsATS.201905-394OC (PMC7175980; doi:10.1513/AnnalsATS.201905-394OC)
Supplement: Supplements [file AnnalsATS.201905-394OC.html]

Temporal Factors and Missed Doses of Tuberculosis Treatment. A Causal Associations Approach to Analyses of Digital Adherence Data | Annals of the American Thoracic Society

- disclosures.pdf (2 MB)
- stagg\_data\_supplement.pdf (790 KB)
