# Supplementary material for: Temporal Factors and Missed Doses of Tuberculosis Treatment. A Causal Associations Approach to Analyses of Digital Adherence Data
Source: Ann Am Thorac Soc. 2020 Apr;17(4):438–49. doi: 10.1513/AnnalsATS.201905-394OC (PMC7175980; doi:10.1513/AnnalsATS.201905-394OC)
Supplement: Supplements [file AnnalsATS.201905-394OC_stagg_data_supplement.pdf]

ONLINE SUPPLEMENT

Temporal factors and missed doses of tuberculosis treatment: a causal associations approach to analyses of digital adherence data

Helen R. Stagg, James J. Lewis, Xiaoqiu Liu, Shitong Huan, Shiwen Jiang, Daniel P. Chin, Katherine L. Fielding

Additional Methods

Parent study and study population for analysis: additional details

Between 1st June 2011 and 7th March 2012, in the Heilongjiang, Jiangsu, Hunan, and Chongqing provinces of the People's Republic of China, 4,173 eligible pulmonary TB patients placed on the standard six-month anti-tuberculosis regimen were consented to be enrolled in a pragmatic cluster randomized trial of electronic reminders (short message service [SMS] and audio reminders from a medication monitor box) to improve treatment adherence.(1) The thirty-six clusters were rural counties or urban districts within these provinces. In all arms of the study, each month a patient's medication was placed in their medication monitor box by local health service staff. The box captured every date and time on which it was opened. These data were downloaded at the monthly clinic visits, at which new medication was dispensed.

Within the control arm of the trial, participants were managed according to the standard of care of the National TB Control Program (NTP). They received no electronic reminders to take their medications; their treatment was either self-administered, or supervised by family members or health care workers. Further restrictions to be included within the cohort analyzed in this study were: having no power outage problems with the medication monitor (resulting in box opening not being recorded), no hospital inpatient stay greater than three days, no pausing/stoppage of treatment due to side effects, and being enrolled into the trial on the same day as TB registration such that treatment had not already started and thus all doses could be captured.

Measuring and defining adherence to treatment: interpreting data from the medication monitor

Data from the medication monitor box were interpreted as follows. If the box was opened at least once within each two-day dosing window this was recorded as adherence, together with the date. If the box was not opened within this period no adherence data were recorded

by the monitor. To document non-adherence at any point, we inferred the dates of missed doses and thus non-adherence when the monitor did not record being opened. Data from the first 180 days were used in the analysis; data on doses taken after this period were not used.

Temporal exposures and confounding: additional information about categorization

The following temporal measures were calculated from the medication monitor data: 1) the day of the week on which each expected dose of medication fell, 2) the treatment month of the dose (expected doses 1-15 fell in month 1, etc.), 3) whether the expected dose fell on a Chinese national holiday, and finally 4) the first 30 expected doses were assigned to the initiation phase of treatment and the last 60 doses to the continuation phase. The latter division is the norm for TB treatment; in the initiation phase four drugs are used for two months, in the continuation phase two drugs are used for four months. The Chinese national holidays considered were New Year (January), Chinese New Year (January), Tomb Sweeping Day (April), Labor Day (April/May), The Dragon Boat festival (June), mid-autumn festival (September), and National Day (October).

Levels of suboptimal dosing implementation in the initiation phase and month 1 were also calculated and categorized.

Associations between temporal factors and suboptimal dosing implementation: detailed methodology used

Adherence data were included for each patient up until the last dose taken before a permanent stoppage of treatment (discontinuation) or the 180-day end point of the regimen, whichever was sooner. Doses after the 180-day (90 dose) point were considered when assessing discontinuation, however (see Methods: Measuring and defining adherence to treatment).

Our analyses focused on the temporal factors of weekends, national holidays and, separately, either the initiation-continuation phase transition (Model 1) or treatment months (Model 2). Having drawn a directed acyclical graph (DAG), the following were deemed *a priori* confounders: age, sex and rural-urban. Assessing the effect of treatment months in place of the initiation-continuation phase transition was decided upon *ad hoc*, after examining our line graphs.

When building our main adjusted model (Model 1) the following factors were additionally considered from the DAG. On the basis of biological plausibility age, treatment month and distance to tuberculosis (TB) clinic were selected *a priori* for an assessment of goodness of fit as linear or categorical variables. Effect estimates across strata were compared and likelihood ratio tests (LRTs) undertaken. Additionally, interactions between national holidays/weekends and the initiation-continuation phase transition or treatment month were tested for using LRTs. The impact of adding a random effect for treatment month and initiation-continuation phase, such that their effect varied between individuals, was also assessed using LRTs.

Model 1 was adapted by adjusting for different sets of potential confounders in place of rural-urban in addition to the *a priori* confounders. These potential confounders could not all be simultaneously assessed due to collinearity. The confounder sets were: distance from home to local TB clinic (Model 1A), medical insurance (Model 1B), occupation (Model 1C), rural-urban and education level (Model 1D), rural-urban and total household income in the last year (Model 1E).

A sensitivity analysis was conducted to examine the impact of potential clustering by county/district, by including this variable as a fixed effect in place of rural-urban (Model 1F). It could not be included as a random effect, due to the small number of counties/districts.

Associations between early suboptimal dosing implementation and time to discontinuation: detailed methodology used

Non-adherence due to suboptimal dosing implementation was categorized into three levels: <80%, 80-89% and ≥90%. The same *a priori* confounders and rural-urban variable were adjusted for as previously, on the basis of a DAG. The validity of the proportional hazards assumption was assessed using a likelihood ratio test (LRT) for an interaction between time and the main exposure of interest.

A sensitivity analysis was also conducted for Models 3 and 4 using a fixed effect for county/district in place of rural-urban status (Models 3F and 4F). An additional analysis excluded individuals who discontinued during the last three doses (approximately a week), in order to focus on earlier time points of discontinuation (Models 3G and 4G).

Table E1. Comparison of baseline characteristics between individuals included in and excluded from the analysis cohort

p-values from X^2 tests.

Exposure variables	Analysis dataset				p-value
	Included	Col. %	Excluded	Col. %	
**Overall**	**780**	**70.7**	**324**	**29.3**	
Sex					p=0.09
Male	535	68.6	239	73.8	
Female	245	31.4	85	26.2	
Age categorised (years)					p=0.73
<30	230	29.5	103	31.8	
30-39	128	16.4	49	15.1	
40-59	303	38.8	129	39.8	
60+	119	15.3	43	13.3	
Occupation					p=0.28
Students	32	4.1	22	6.8	
Worker	74	9.5	27	8.3	
Migrant Worker	74	9.5	24	7.4	
Farmer	384	49.2	156	48.1	
Unemployed/Houseworker	63	8.1	22	6.8	
Other	153	19.6	73	22.5	
Educational level					p=0.61
Illiterate	60	7.7	21	6.5	
Lower middle school	494	63.3	199	61.4	
Upper middle school	130	16.7	64	19.8	
University or more	96	12.3	40	12.3	
Total household income in last calendar year (RMB)					p=0.92
≥20,000	320	41.0	134	41.4	
<20,000	460	59.0	190	58.6	
Medical insurance					p=0.56
Rural co-op	500	64.1	210	64.8	
Urban workers	92	11.8	42	13.0	
No insurance	132	16.9	56	17.3	
Other	56	7.2	16	4.9	
Marital status					p=0.41
1st marriage	551	70.6	219	67.6	
Unmarried	184	23.6	80	24.7	
Other	45	5.8	25	7.7	
County					p<0.001
Baiquan	100	12.8	25	7.7	
Yilan	103	13.2	20	6.2	
Rugao	78	10.0	40	12.3	
Jianhu	80	10.3	39	12.0	
Miluo	85	10.9	33	10.2	
Yueyanglou	81	10.4	38	11.7	
Fengjie	70	9.0	60	18.5	
Shapingba	79	10.1	52	16.0	
Jiangbei	104	13.3	17	5.2	
Rural/urban					p=0.79
Rural	516	66.2	217	67.0	
Urban	264	33.8	107	33.0	

Table E1. continued

Exposure variables	Analysis dataset Included	Col. %	Excluded	Col. %	p-value
Residence					p=0.97
Living in place of household registration	658	84.4	273	84.3	
Not living in place of household registration	122	15.6	51	15.7	
Distance from home to local TB clinic (km)					p=0.003
<10	188	24.1	69	21.3	
10-29	309	39.6	117	36.1	
30-39	149	19.1	51	15.7	
>=40	134	17.2	87	26.9	

Table E2. Adjusted logistic regression of the association between temporal factors and suboptimal dosing implementation, adjusting for different confounder sets

Adjusted models of the association between the temporal factors weekend, national holidays, and treatment phase and the outcome of non-adherence due to suboptimal dosing implementation. All models derive from Model 1. Each adjusts for the other temporal factors listed in the relevant stratum of the table plus age, sex and: distance from home to local TB clinic rather than rural-urban (Model 1A), medical insurance rather than rural-urban (Model 1B), occupation rather than rural-urban (Model 1C), both rural-urban and education level (Model 1D), rural-urban and total household income in last calendar year (Model 1E), county/district rather than rural-urban (Model 1F). 62,893 doses from 780 individuals in the control arm of the original trial included. Random effect modelled on the initiation-continuation phase variable. Age and distance to TB included as linear variables, where relevant. CI- confidence interval, OR- odds ratio, TB- tuberculosis.

Temporal factor		OR (95% CI)
MODEL 1A		
Weekend		p<0.001
	Weekday	baseline
	Weekend	1.14 (1.08-1.20)
National holidays		p<0.001
	No	baseline
	Yes	1.52 (1.39-1.65)
Phase		p<0.001
	Initiation	baseline
	Continuation	3.07 (2.68-3.51)
MODEL 1B		
Weekend		p<0.001
	Weekday	baseline
	Weekend	1.14 (1.08-1.20)
National holidays		p<0.001
	No	baseline
	Yes	1.52 (1.39-1.65)
Phase		p<0.001
	Initiation	baseline
	Continuation	3.07 (2.68-3.51)
MODEL 1C		
Weekend		p<0.001
	Weekday	baseline
	Weekend	1.14 (1.08-1.20)
National holidays		p<0.001
	No	baseline
	Yes	1.52 (1.39-1.65)
Phase		p<0.001
	Initiation	baseline
	Continuation	3.08 (2.69-3.53)
MODEL 1D		
Weekend		p<0.001
	Weekday	baseline
	Weekend	1.14 (1.08-1.20)
National holidays		p<0.001
	No	baseline
	Yes	1.52 (1.39-1.65)
Phase		p<0.001
	Initiation	baseline
	Continuation	3.06 (2.67-3.50)
MODEL 1E		
Weekend		p<0.001
	Weekday	baseline
	Weekend	1.14 (1.08-1.20)
National holidays		p<0.001
	No	baseline
	Yes	1.52 (1.39-1.65)
Phase		p<0.001
	Initiation	baseline
	Continuation	3.07 (2.69-3.52)

Table E2. continued

Temporal factor		OR (95% CI)
MODEL 1F		
Weekend		p<0.001
	Weekday	baseline
	Weekend	1.14 (1.08-1.20)
National holidays		p<0.001
	No	baseline
	Yes	1.52 (1.39-1.65)
Phase		p<0.001
	Initiation	baseline
	Continuation	3.14 (2.74-3.60)

Table E3. Adjusted odds ratios for the association between suboptimal dosing implementation and the initiation-continuation phase transition, stratified by county

		Phase	
		Initiation	Continuation
**County**	Baiquan	baseline	5.12 (3.69-7.11)
	Yilan	baseline	2.64 (1.88-3.71)
	Rugao	baseline	2.12 (1.44-3.13)
	Jianhu	baseline	2.63 (1.83-3.78)
	Miluo	baseline	3.31 (2.28-4.78)
	Yueyanglou	baseline	2.48 (1.65-3.73)
	Fengjie	baseline	4.18 (2.71-6.44)
	Shapingba	baseline	4.05 (2.75-5.98)
	Jiangbei	baseline	2.44 (1.73-3.45)

Adjusted regression of the association between non-adherence due to suboptimal dosing implementation and the initiation-continuation phase transition (Model 1), stratified by county (Model 1G). 62,893 doses from 780 individuals from the control arm of the original trial included. The stratum-specific ORs are adjusted for weekends, holidays, age, sex and county. Random effect modelled on the initiation-continuation phase variable. Age modelled as a linear variable. Results per cell presented as OR (95% CI). CI- confidence interval, OR- odds ratio

Table E4. Adjusted odds ratios for the association between suboptimal dosing implementation and holidays, stratified by county

		Holiday	
		No	Yes
**County**	Baiquan	baseline	0.94 (0.75-1.18)
	Yilan	baseline	1.49 (1.18-1.88)
	Rugao	baseline	1.43 (1.08-1.89)
	Jianhu	baseline	1.39 (1.10-1.77)
	Miluo	baseline	1.77 (1.37-2.28)
	Yueyanglou	baseline	1.57 (1.17-2.11)
	Fengjie	baseline	1.41 (1.02-1.94)
	Shapingba	baseline	1.75 (1.35-2.27)
	Jiangbei	baseline	2.37 (1.89-2.96)

Adjusted regression of the association between non-adherence due to suboptimal dosing implementation and holidays (Model 1), stratified by county (Model 1H). 62,893 doses from 780 individuals from the control arm of the original trial included. The stratum-specific ORs are adjusted for weekends, initiation-continuation phase transition, age, sex and county. Random effect modelled on the initiation-continuation phase variable. Age modelled as a linear variable. Results per cell presented as OR (95% CI). CI- confidence interval, OR- odds ratio

Table E5. Adjusted Cox regression models of the association between early suboptimal dosing implementation and discontinuation- sensitivity analysis

Sensitivity analysis of the association between suboptimal dosing implementation in the initiation phase or month 1, and discontinuation. Model 3F examines the association between suboptimal dosing implementation in the initiation phase and discontinuation, adjusting for age, sex and county/district (as opposed to rural-urban in Model 3). It excludes individuals who discontinued in the initiation phase, leaving 740. Model 3G examines the association between suboptimal dosing implementation in the initiation phase and discontinuation whilst excluding individuals who discontinued after dose 86 (688 people in the model) and adjusts for the same confounders as Model 3. Model 4F examines the association between suboptimal dosing implementation in month 1 and discontinuation, adjusting for age, sex and county/district (as opposed to rural-urban in Model 4). It excludes individuals who discontinued during month 1, leaving 775. Model 4G examines the association between suboptimal dosing implementation in month 1 and discontinuation whilst excluding individuals who discontinued after dose 86 (723 people in the model) and adjusts for the same confounders as Model 4. Age modelled as a linear variable in all models. CI- confidence interval.

Temporal factor	Hazard ratio (95% CI)
MODEL 3F	
Initiation phase adherence	p=0.001
≥90%	baseline
80-<90%	1.17 (0.74-1.84)
<80%	2.14 (1.46-3.14)
MODEL 3G	
Initiation phase adherence	p=0.001
≥90%	baseline
80-<90%	1.28 (0.78-2.09)
<80%	2.40 (1.59-3.62)
MODEL 4F	
Month 1 adherence	p=0.002
≥90%	baseline
80-<90%	1.53 (1.05-2.22)
<80%	2.06 (1.33-3.16)
MODEL 4G	
Month 1 adherence	p=0.001
≥90%	baseline
80-<90%	1.51 (1.00-2.28)
<80%	2.39 (1.52-3.78)

REFERENCES

1. Liu X, Lewis JJ, Zhang H, Lu W, Zhang S, Zheng G, et al. Effectiveness of Electronic Reminders to Improve Medication Adherence in Tuberculosis Patients: A Cluster-Randomised Trial. *PLoS Med* 2015;12:e1001876.

FIGURE LEGENDS

Figure E1. Flow chart of participants

Flow chart documenting participation from the original trial to this study. Side effects could lead to temporary or permanent medication stoppage; in either instance, adherence data were no longer collected.

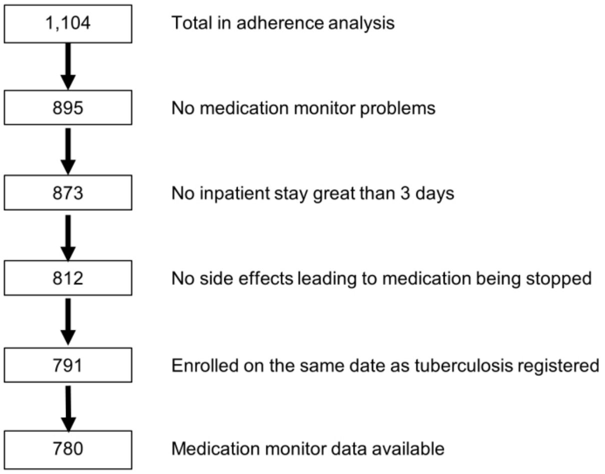

Figure E1. Flow chart of participants
Flow chart documenting participation from the original trial to this study. Side effects could lead to temporary or permanent medication stoppage; in either instance, adherence data were no longer collected.

190x254mm (300 x 300 DPI)
